# Temporal and Spatial Dynamics of Rodent Species Habitats in the Ordos Desert Steppe, China

**DOI:** 10.3390/ani15050721

**Published:** 2025-03-03

**Authors:** Rui Hua, Qin Su, Jinfu Fan, Liqing Wang, Linbo Xu, Yuchuang Hui, Miaomiao Huang, Bobo Du, Yanjun Tian, Yuheng Zhao, Manduriwa

**Affiliations:** 1 Inner Mongolia Key Laboratory of Grassland Protection Ecology, Grassland Research Institute, Chinese Academy of Agricultural Science, Hohhot 010010, China; huarui_gsau@163.com (R.H.);; 2 Ordos Forestry and Grassland Bureau, Ordos 017000, China; cyzsuqin@126.com (Q.S.);; 3 Otog Banner Forestry and Grassland Bureau, Ordos 016100, China; 4 Otog Front Banner Forestry and Grassland Ecological Conservation Center, Ordos 016100, China

**Keywords:** climate change, maxent model, habitat suitability, bioclimatic variables, normalized difference vegetation index

## Abstract

Climate change is driving the restructuring of global biological communities. As a species sensitive to climate change, studying the response of small rodents to climate change is helpful to indirectly understand the changes in ecology and biodiversity in a certain region. Here, we use the MaxEnt (maximum entropy) model to predict the distribution patterns, main influencing factors, and range changes of various small rodents in the Ordos desert steppe in China under different climate change scenarios in the future (2050s: average for 2041–2060). The results show that when the parameters are FC = LQHPT, and RM = 4, the MaxEnt model is optimal and AUC = 0.833. We found that NDVI (normalized difference vegetation index), Bio 12 (annual precipitation), and TOC (total organic carbon) are important driving factors affecting the suitability of the small rodent habitat distribution in the region. At the same time, the main influencing factors were also different for different rodent species. We selected 4 dominant species for analysis and found that, under the situation of future climate warming, the high-suitability habitat area of *Allactaga sibirica* and *Phodopus roborovskii* will decrease, while that of *Meriones meridianus* and *Meriones unguiculatus* will increase. Our research results suggest that local governments should take early preventive measures, strengthen species protection, and respond to ecological challenges brought about by climate change promptly.

## Simple Summary

The effects of climate change, human disturbance, and other environmental factors on the distribution and habitat suitability of small rodents in the Ordos Basin were studied using the MaxEnt model. The results showed that NDVI, average annual precipitation, and soil organic carbon content were the key factors affecting the habitat suitability of rodents in the Ordos Basin. Anticipated habitat changes could disrupt local ecosystems and increase the risk of zoonotic diseases such as plague due to changes in rodent populations. Our study therefore highlights the urgent need for local governments to take early preventive measures, strengthen priority species monitoring efforts, and develop adaptation strategies to mitigate the adverse impacts of climate change on biodiversity and public health.

## 1. Introduction

Future global warming will significantly impact the spatial distribution of species, leading to various adaptations to the changing environmental conditions [1,2,3]. A common consequence of such spatial redistribution is a reduction in species’ distribution ranges [4]. Grasslands, as a crucial global ecosystem, are highly sensitive to climate change and are experiencing severe degradation worldwide [5]. In many regions of Asia, climate change combined with poor grassland management has led to a notable biodiversity crisis [6], particularly affecting small rodents that are highly sensitive to climate change and human disturbances. These rodents are key to maintaining grassland biodiversity, playing roles as predators, prey, competitors, and mutualists, and sustaining ecological balance through their interactions with other species. As important components of the ecosystem and indicators of environmental changes, they are suitable subjects for studying the effects of climate change, vegetation, topography, and human interference on animals. This group is especially sensitive to the influence of climatic conditions on diversity and species composition in different natural conditions [7,8].

The Ordos Plateau of Inner Mongolia, China, holds significant ecological functions and economic value. With a northwest–high, southeast–low terrain and diverse landforms, it features unique climate characteristics, including strong continentality, low and uneven precipitation, abundant sunlight, relatively strong winds, and intense evaporation, all of which have given rise to vast desert steppes. These grasslands not only shape the landscape but also provide habitats for a diverse range of highly adaptive small rodents. Their presence plays a crucial role in the ecosystem, influencing soil structure and plant regeneration, and contributing to the delicate ecological balance of the desert steppe [9].

However, the fragile ecological environment of Ordos, compounded by its inherent vulnerabilities and the pressures of economic development, has led to significant ecological challenges, including soil erosion and desertification. These issues have worsened in recent years due to global environmental changes—such as climate change, altered precipitation, and rising CO_2_ levels—combined with human disturbances, significantly impacting the biodiversity and ecological functions of desert grassland ecosystems. As a result, the desert steppe of Ordos has suffered extensive degradation, compromising the structure and the function of the ecosystem [10].

Rodent populations are important biological disturbance factors in the desert grassland ecosystem of this region, and they are closely linked to its overall health [11]. However, there is limited research on the spatial distribution and habitat suitability of small rodents in this area. Understanding the potential distribution and habitat suitability of small rodents in this critical region is essential for the conservation and management of the grassland ecosystem in the Ordos, as it not only aids in pest control and biodiversity conservation but also offers valuable insights into how small rodents in arid and semi-arid regions may respond to climate change and human disturbances in the future.

Currently, most ecologists tend to use species distribution models (SDMs) to predict habitat suitability and the potential distributions of various species [12]. Based on niche theory, SDMs quantify the relationship between environmental data and species distribution data to infer the ecological requirements of species, expressing the probability of species presence in a particular habitat [13]. Therefore, they are also known as ecological niche models [14]. Among these, MaxEnt is considered one of the most stable and effective SDMs, and it has been proven to provide accurate predictions for past and future climate scenarios [15,16,17]. It is widely used for predicting the potential distributions of species under climate change. Additionally, with the application of new technologies and a deeper understanding of climate change patterns, the WorldClim database (https://www.worldclim.org, accessed on 1 July 2024) increasingly aligns future climate data simulations with actual monitoring [18].

Based on the maxent model, this study simulated and analyzed the relationship between rodent distribution, environmental factors, and climate change in the Ordos desert steppe in an attempt to provide basis for biodiversity conservation and planning decisions in the grassland ecosystem. We hypothesized that climate and environmental factors significantly influenced the distribution of rodent species in the Ordos desert steppe. To achieve the research objectives, the study was carried out through the following approaches:

1. Clarify the potential suitable habitats and areas for rodent species in the Ordos 96 desert steppe.
2. Identify the main environmental factors affecting the distributions of rodent species in the Ordos desert steppe and analyze their responses to these factors.
3. Investigate the changes in the distribution range and area of rodents in the Ordos desert steppe under the condition of climate change, and further clarify the severity of the rodent problem in the study area, to provide the basis for subsequent management and control.

## 2. Materials and Methods

### 2.1. Species Distribution and Environmental Data Acquisition

#### 2.1.1. Species Distribution Data

The geographical distribution data of rodent species in this study were obtained via a field investigation (Ordos, 34° N–40° N, 104° E–112° E) that has been monitoring this area for five consecutive years (2018–2022), ensuring a comprehensive assessment of rodent presence in the surveyed regions. We used peanuts as bait and set traps in each standard survey cell, with 3 replicates per cell. To ensure independence, the distance between each replicate was set to be more than 300 m. In each replicate, a total of one hundred traps were placed. These traps were arranged in four rows, with 25 traps in each row. The distance between rows was 20 m, and the distance between adjacent traps was 5 m; the traps were designed to capture live rodents. Once captured, each rodent was individually tagged with red animal-marking solution and then released. The traps were set continuously for two days, and we conducted daily inspections. The most recent set of captured data used in this study is from 2022, where a total of 8937 traps were deployed across different banners in the Ordos desert steppe, of which 8730 yielded valid results. We captured a total of 700 small rodents from 9 species and selected 4 dominant species for analysis. Trap coordinates were recorded using GPS.

**Figure 1:**
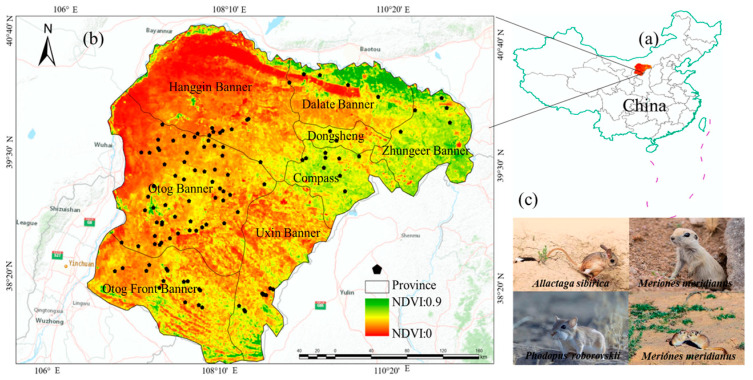
Main distribution points of all rodent species. (**a**) is the Chinese region, (**b**) is the study area, and (**c**) is part of the rodents in the study area.

To address potential overfitting resulting from environmental biases and model performance inflation due to spatial clustering, spatial rarefaction was conducted using the SDMtool box toolkit in ArcGIS 10.2 [2,19]. A total of 187 survey points, spaced at intervals greater than 1 km, were retained (Figure 1, Table S1). Distribution data with latitude and longitude were saved in “csv” format for later use.

#### 2.1.2. Environment Data (19 in Total)

In this study, we selected four distinct environmental factors to assess their impact on the habitat suitability of rodents inhabiting desert steppe, including climate, vegetation, topography, and anthropogenic disturbance. In terms of climate variables, 19 bioclimatic variables were chosen from both recent historical (1970–2000) and future (2050s: average for 2041–2060) climate scenarios (http://worldclim.org, accessed on 1 July 2024; 1 km spatial resolution) [20]. For small rodents, the 2050s offer higher predictive reliability compared to more distant future time points. This period better reflects the potential impact of current climate change trends on their distribution and provides a relatively clear medium-term forecast. The general circulation models (GCMs) of the Shared Socio-economic Pathway (SSP) scenarios, developed by the Intergovernmental Panel on Climate Change’s (IPCC) Coupled Model Intercomparison Project Phase 6 (CMIP6), were employed to assess potential climate shifts in the future [21]. We selected two different pathways of CO_2_ emissions, namely Bc26 (low greenhouse gas emissions) and Bc45 (higher greenhouse gas emissions). These projections were based on the BCC-CSM2-MR (the Beijing Climate Center Climate System Model) climate model from the Chinese National Climate Centre, which has a high simulation capability in China [22,23,24]. The data were sourced from the WorldClim Global Climate Database (http://worldclim.org, accessed on 1 July 2024; 1 km spatial resolution).

#### 2.1.3. Vegetation Data (2 in Total)

Landsat 8 images from July were downloaded and cropped to the study area. NDVI was calculated using the formula to assess vegetation cover. Vegetation type data were provided by the National Cryosphere Desert Data Center (http://www.ncdc.ac.cn, accessed on 1 July 2024).

#### 2.1.4. Topographic Variables and Anthropogenic Variables (8 in Total)

Elevation, slope, and aspect information were obtained from the Geographic Spatial Data Cloud (https://www.gscloud.cn, accessed on 1 July 2024). Human disturbance was quantified using the Human Footprint Index (HFI), the data for which were acquired from the Figshare website (https://figshare.com/, accessed on 1 July 2024). The soil data were sourced from the HWSD Global Soil Database (http://www.fao.org/, accessed on 1 July 2024, HWSD V1.2; 1 km spatial resolution). We selected pH, TOC (total organic carbon), sand content, and bulk density from the 0–20 cm soil layer for analysis.

### 2.2. Data Analysis

Environmental variation in the geographical range could be indicated well by 1 km resolution variables. The 1 km resolution bioclimatic data of WorldClim are accessible and represent a rational choice. Therefore, we designated the spatial resolution of all data as 1 km. The above environmental variables were converted to an “asc” format based on ArcGIS 10.2 software. To ensure model accuracy, we conducted an analysis of 19 climatic variables based on field survey points before modeling. The aim was to remove variables with strong collinearity by calculating Spearman correlation coefficients, and variables with high correlation (r > 0.8) were excluded [25,26] (Figure S1). Finally, 16 environmental factors were selected to predict the potential area (Table 1).

### 2.3. Model Setting and Evaluation

Unoptimized model predictions may exhibit a significant tendency toward overfitting [27]. To optimize the MaxEnt model, the regularization multiplier (RM) ranges from 0.5 to 4, with an increase of 0.5 per run [28]. The feature combination is set to 6, namely L, LQ, H, LQH, LQHP, and LQHPT [3]. It is necessary to invoke the ENMeval package of R 4.2.3 software to test the above 48 parameter combinations, and also to check the complexity and fit degree of the model according to deltaAICc (Akaike information criterion, corrected), AUCdiff (different between training and testing AUC), and OR10 (10% training omission rate) to measure the degree to which models overfit species distribution points [29,30]. To assess the performance of our Maxent model in predicting the habitat suitability for rodents, we utilized the ROC (receiver operating characteristic) area under the curve (AUC) and true skill statistic (TSS) to evaluate the prediction accuracy of the MaxEnt model [31]. The AUC value is a widely accepted measure of model accuracy, with higher values indicating greater precision in predictions. Model performance, based on AUC values, is categorized as follows: failing (0.5–0.6), poor (0.6–0.7), fair (0.7–0.8), good (0.8–0.9), and excellent (0.9–1.0) [32,33]. The TSS (true skill statistic) value ranges from −1 to 1, with values closer to 1 indicating better predictive performance. Therefore, higher TSS values indicate more reliable and accurate predictions [34]. The regularization multiple is used to control the complexity of the model. We choose the regularization multiple with the highest average AUC as the optimal value. By optimizing regularization multiples and evaluating MaxEnt models using AUC and TSS metrics, we can improve model accuracy and prediction performance [35,36].

### 2.4. Classification of Potentially Suitable Area

The average value of MaxEnt model output results was imported into ArcGIS. The natural fracture method (Jenks) was used to reclassify the simulated suitable area results and divide them into four suitable levels: an unsuitable area (0–0.2), a sub-suitable area (0.2–0.4), a medium-suitability area (0.4–0.6), and a highly suitable area (0.6–1) [37]. The projection coordinate system chosen in this study is the WGS 1984 UTM 48N region and, finally, the proportion and area of each level of suitability area are calculated.

## 3. Results

### 3.1. Community Composition of Rodent Species

**Figure 2:**
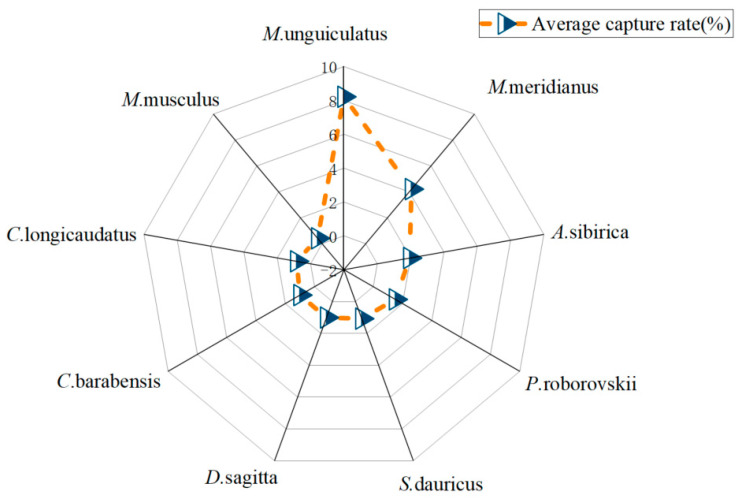
The average capture rate of different rodents in the study area.

The dominant species of rodent communities play a vital role in the ecosystem. Through field investigation, a total of 700 rodents were captured, comprising nine species: *Meriones unguiculatus* (Milne-Edwards, 1867), *Spermophilus dauricus* (Brandt, 1843), *Meriones meridianus* (Pallas, 1773), *Allactaga sibirica* (Forster, 1778), *Cricetulus barabensis* (Pallas, 1773), *Phodopus roborovskii* (Satunin, 1903), *Cricetulus longicaudatus* (Milne-Edwards, 1867), *Mus musculus* (Linnaeus, 1758), and *Dipus sagitta* (Pallas, 1773). Statistical analysis identified *M. unguiculatus*, *M. meridianus*, *A. sibirica*, and *P. roborovskii* as the dominant species in the area (Figure 2).

### 3.2. Simulation of Suitable Habitats for Rodentia and Extraction of Key Influencing Factors

#### 3.2.1. Optimization and Accuracy Evaluation of the Model

**Figure 3:**
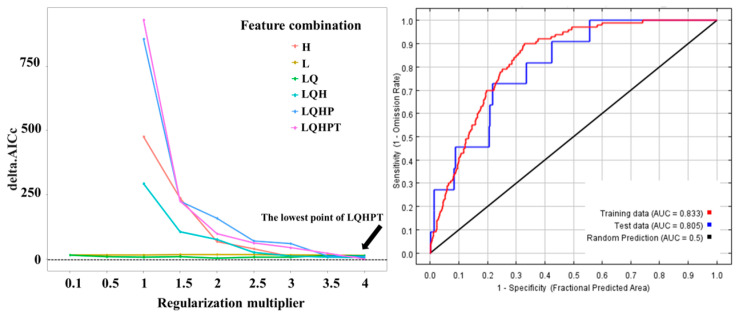
Results of ENMeval of Rpackage and the prediction results of ROC by MaxEnt model.

According to the model optimization results, the optimal model combination was selected from 48 parameter combinations. When RM is 4, FC is LQHPT, and deltaAICc = 0, the model is optimal (Figure 3). After verification, the TSS value is 0.713, indicating that the model has a good prediction effect. It can be seen from the characteristic curve that under the current baseline climate conditions, the prediction accuracy of the potential distribution of small rodents is high, with a training dataset AUC of 0.833 and the test dataset AUC of 0.805 (Figure 3), indicating good model performance (AUC > 0.8).

#### 3.2.2. Simulation of Suitable Habitats for Rodentia and Extraction of Key Influencing Factors

To clarify the impact of environmental factors on the distribution and survival of small rodents, this study first treats all rodent species collectively for a comprehensive analysis of suitable habitats. Subsequently, the distribution of dominant species within the study area is examined individually.

**Figure 4:**
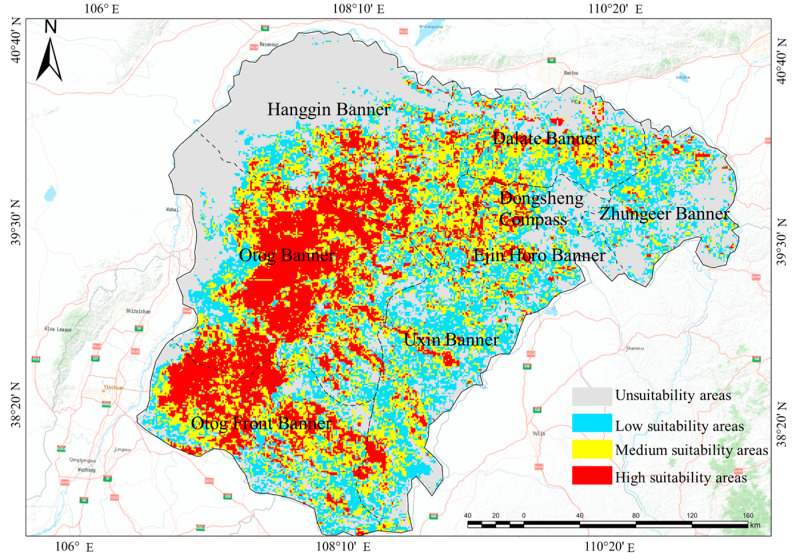
Distribution of suitable and non-suitable areas for small rodents.

**Figure 5:**
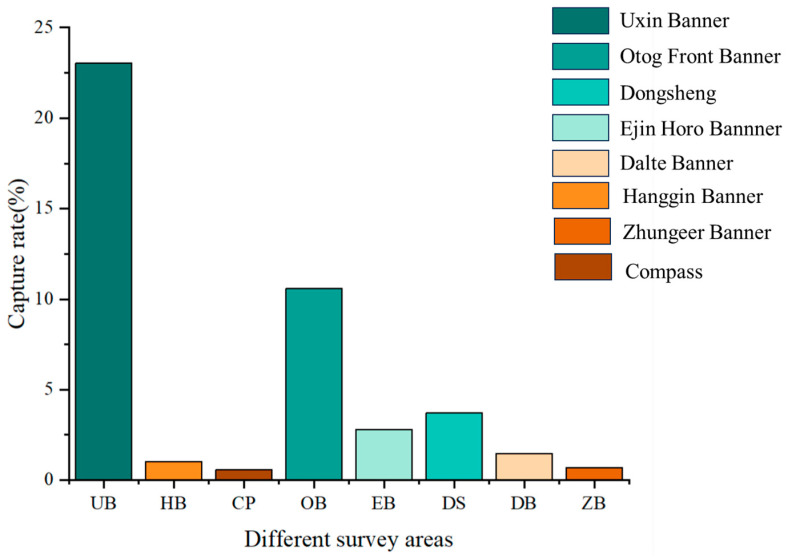
Total capture rates of rodent communities in different districts of Ordos.

The MaxEnt model prediction results indicate that, under baseline climate conditions, the primary suitable habitats for small rodents are mainly located in the western and southern parts of the Ordos desert steppe (Figure 4), covering 4.44% of the total area. The moderately suitable habitats are primarily found at the edges of the highly suitable areas. Highly suitable areas account for 20.5% of the city’s total area, while non-suitable areas cover 30.9% (Table 2). Actual survey results show that rodent community capture rates are highest in Wushen Banner, reaching 23.38%, followed by Otog Front Banner at 10.76%, and Otog Banner at 7.25%. These actual survey results are consistent with the model predictions (Figure 5).

#### 3.2.3. Analysis of Environmental Variable Contributions and Response Curves

**Figure 6:**
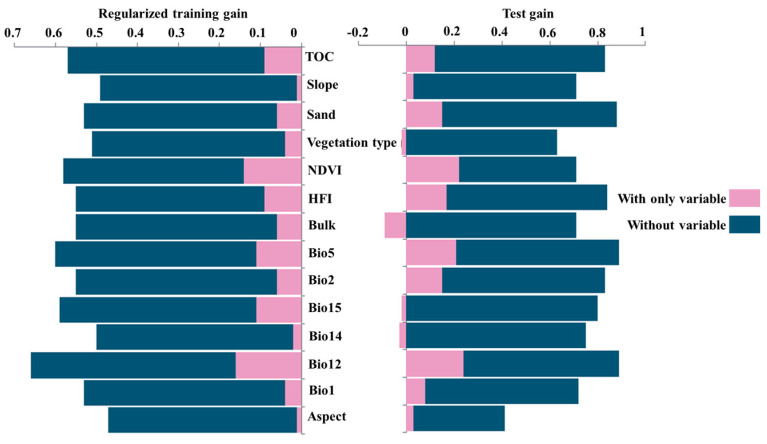
Results of the jackknife test for the environmental variables predicted.

The model results indicate that NDVI, Bio12, and TOC are key drivers of habitat suitability for small rodents in this region, with a cumulative importance value of 56.1% (Table 3). The jackknife test of the 15 environmental variables also shows that, in the single-variable model, the environmental variables with the highest gains in regularized training, testing, and AUC values are NDVI and Bio12. Bio5 also contributes significantly, indicating that these factors have high predictive capabilities (Figure 6). Overall, the primary factor affecting the geographic distribution of small rodents in this region is the vegetation condition, followed by significant influences from precipitation and total organic carbon content.

**Figure 7:**
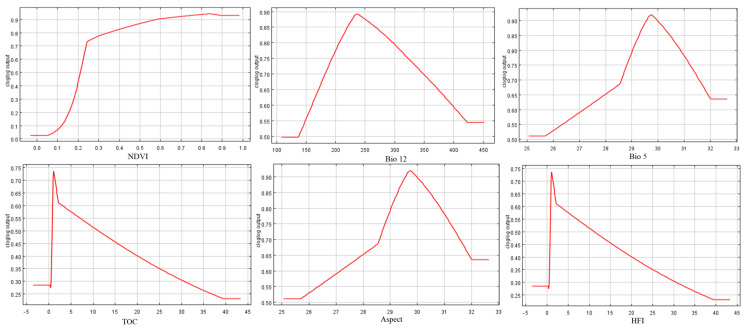
Response curves for the main environmental factors in Maxent modeling.

**Figure 8:**
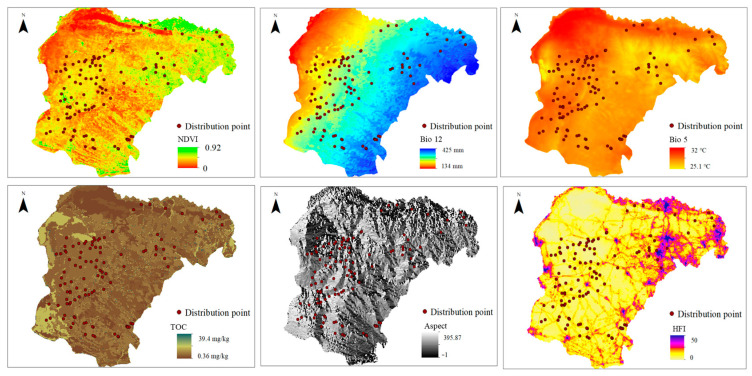
Environmental variables used for modeling.

After extracting the dominant environmental factors for separate analysis, the significant influencing factor ranges for highly suitable distribution areas for small rodents under baseline climate conditions are as follows: NDVI > 0.25; Bio12 of 200–300 mm; Bio5 between 29 °C and 30.8 °C; TOC content at 2 mg/kg. These areas are predominantly found on sunny slopes. Habitat suitability increases with human disturbance values up to a threshold of 6.5, beyond which suitability decreases progressively as HFI increases further (Figure 7 and Figure 8).

### 3.3. Simulation of Suitable Habitats for Dominant Species and Extraction of Key Influencing Factors

#### 3.3.1. Optimization and Accuracy Evaluation of the Model

We also optimized the model parameters for different species. According to the model optimization results, the optimal model combination was selected from 48 parameter combinations. For *A. sibirica*, when RM is 2.5 and FC is L, deltaAICc = 0 is the optimal parameter. For *M. unguiculatus*, when RM is 4 and FC is LQHP, deltaAICc = 0 is the optimal parameter. For *P. roborovskii*, when RM is 2.5 and FC is L, deltaAICc = 0 is the optimal parameter. For *M. meridianus*, when RM is 3 and FC is LHQ, deltaAICc = 0 is the optimal parameter. Based on this, a follow-up study was conducted.

#### 3.3.2. Simulation of Suitable Habitats for Different Dominant Species and Extraction of Key Influencing Factors

The model results (Table 4) show that the mean diurnal temperature range (Bio2), soil sand content, and vegetation type are important driving factors affecting the habitat distribution of *A. sibirica* in the region. Meanwhile, HFI, TOC, and Bio12 are significant driving factors influencing the habitat distribution of *P. roborovskii* in the area. Furthermore, the distribution of *M. unguiculatus* shows a marked response to Bio12, TOC, and Bio1. Lastly, Bio12, bio5, and Bio1 are important driving factors affecting the habitat distribution of *M. meridianus* in the region.

**Figure 9:**
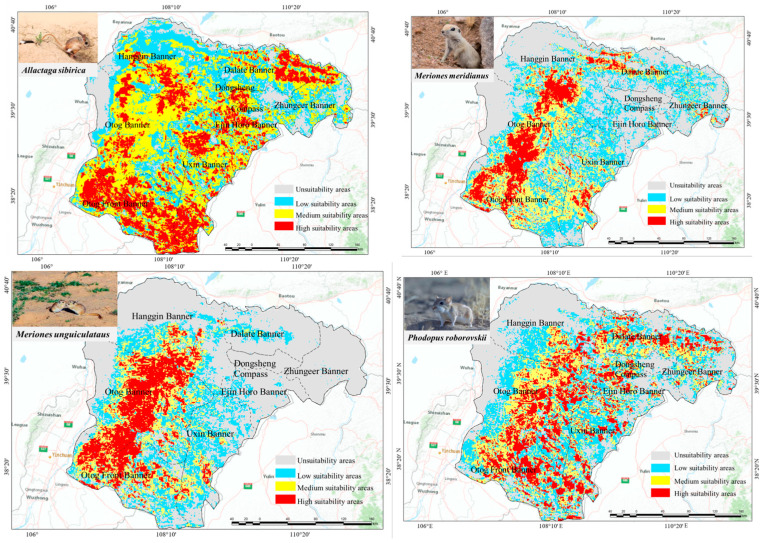
Distribution of suitable and non-suitable areas for different species.

**Figure 10:**
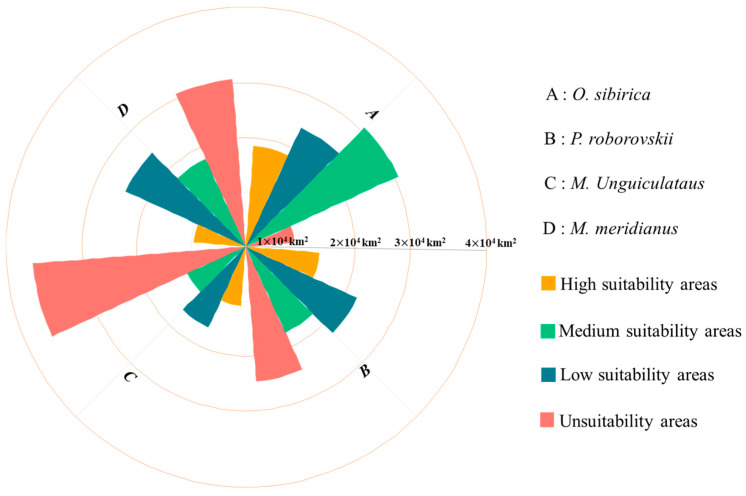
Distribution area statistics of different species under the current climate.

Based on the model results, it is evident that among all rodent species, *A. sibirica* has the largest suitable habitat area, covering approximately 89% of the study area. The suitable habitat for *P. roborovskii* covers about 68% of the study area, that of *M. meridianus* covers around 63%, and finally that of *M. unguiculatus* covers approximately 50% (Figure 9 and Figure 10). Specifically, the main suitable habitat areas for *A. sibirica* are located in the southern and western parts of Ordos; the primary suitable habitats for *M. meridianus* and *M. unguiculatus* are concentrated in Otog Front Banner, Otog Banner, and Hanggin Banner; while the distribution of *P. roborovskii* is more dispersed, with suitable habitats found in various parts of the region. Considering these four species collectively, the regions of Wushen Banner, Otog Banner, and Otog Front Banner have the highest proportions of highly suitable habitats within the city. Actual survey data show that rodent community capture rates are highest in Wushen Banner at 23.38%, followed by Otog Front Banner at 10.76%, and Otog Banner at 7.25%. Therefore, the model simulation results of this study are consistent with actual survey findings.

#### 3.3.3. Prediction of the Potential Distribution of Different Species Under Climate Change

**Figure 11:**
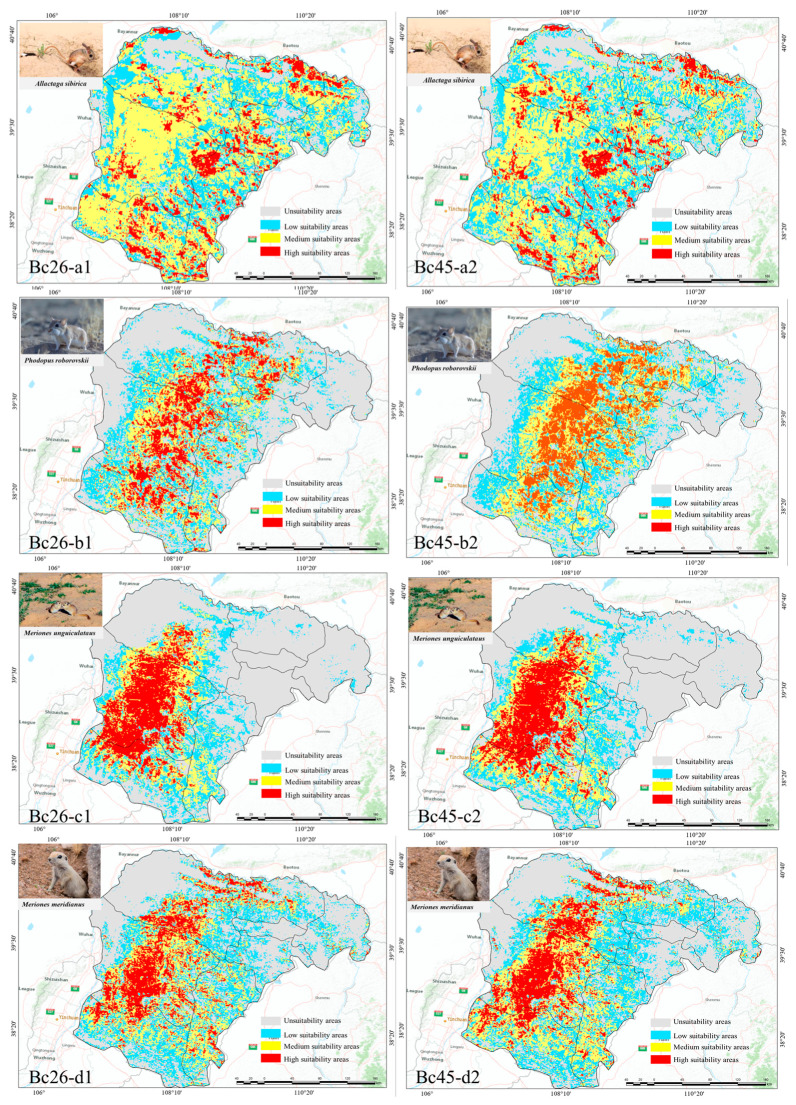
The prediction of the distribution of each species under different climate scenarios.

Under two greenhouse gas emission scenarios from the BCC-CSM2-MR model, the potential distribution areas for different rodent species in the 2050s (2041–2060) were calculated. The results are shown in Figure 11.

**Figure 12:**
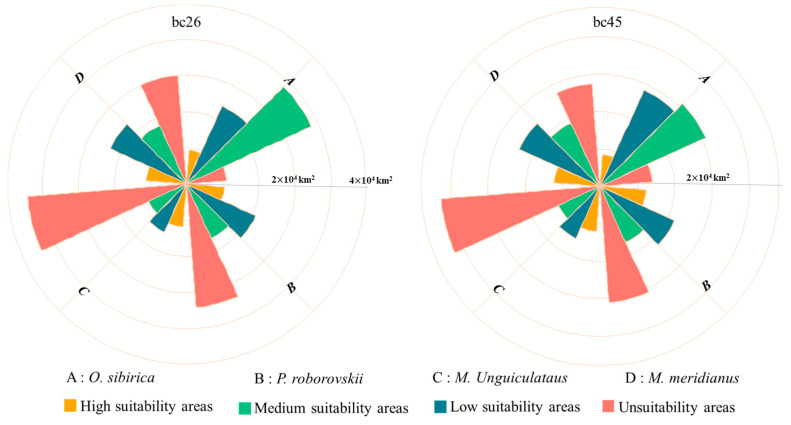
Distribution area statistics of different species in future climate.

Under the two different scenarios projected for the 2050s, the areas of suitable habitats for different rodent species exhibit variations (Figure 12). For *A. sibirica* and *P. roborovskii*, the area of high-suitability habitats declines as the climate warms. Specifically, under the Bc45 emissions scenario, the high-suitability habitat area of *A. sibirica* is reduced by 9856 km^2^ compared with the current climate situation, with a loss percentage of 53.42%. Meanwhile, the high-suitability habitat area of *P. roborovskii* decreases by 1092 km^2^, and the percentage loss is 8.03%.

Conversely, for gerbil species (includes *M. meridianus* and *M. unguiculatus*), the area of high-suitability habitat increases with climate warming. The high-suitability habitat area for *M. unguiculatus* increases by 1271 km^2^ (11.68%) under the Bc45 emissions scenario, and it increases for *M. meridianus* by 2654 km^2^ (27.49%). This result may be due to the strong tolerance of gerbil species to high-temperature environments. In the Bc26 emission scenario, the change trend in high-suitability habitat areas for different rodent species was the same as that of the bc45 emissions scenario.

## 4. Discussion

### 4.1. Effect of Environmental Factors on the Distribution of Rodents

Small-sized rodents respond rapidly and distinctly to environmental changes, making them important indicator species for studying the impacts of climate change on ecosystems. Our research indicates that NDVI, Bio12, and TOC have a significant influence on the habitat suitability of rodents in the Ordos desert steppe. As an indicator of vegetation cover and growth [38], higher NDVI values are associated with a habitat’s suitability of small-sized rodents, as areas with better vegetation can provide more food and shelter. Meanwhile, vegetation affects soil moisture and micro-climate, and factors that can lead to vegetation degradation, such as drought, over-grazing, or land development, will all undermine the survival of rodents [39]. For annual precipitation, the suitability is highest between 200 and 300 mm. Changes in precipitation not only affect the direct availability of water resources but also indirectly impact rodent survival by influencing plant growth and food chain structures [40]. Soil organic carbon content, which affects soil fertility and structure, is a crucial nutrient source for plant growth and provides abundant food resources for small rodents [41]. Soil degradation and a decline in organic matter could directly reduce the suitable habitat area for rodents.

Our research has discovered that moderate human disturbance can improve the habitat suitability of desert rodents [42], and certain human activities may enhance habitat usability through means such as providing food resources and reducing the number of predators [43]. However, once human disturbance becomes excessive, it will reduce habitat suitability. Wan et al. discovered that human disturbance and climate warming are closely related to the decline in the population of most rodent species [44], and the decline is more pronounced in areas severely affected by human activities such as urban and farmland areas. Li et al. also points out that excessive human activities can damage the natural environment, having a negative impact on the habitats of desert rodents and their spatial utilization [45]. In conclusion, moderate human activities can improve habitats, while excessive interference can lead to severe damage. Against the backdrop of global environmental changes, maintaining a proper balance is of great significance for the protection of these species and the stability of ecosystems.

Different rodent species are affected by various key factors. For *A. sibirica*, significant drivers include Bio2, TOC, and the vegetation type. As a nocturnal species active at dusk, the mean diurnal range influences their thermoregulation and activity patterns. Their burrows are typically built in more solid soils, and soil sand content affects habitat suitability and food resource availability. Moreover, the vegetation type is closely associated with the species’ preferences for food sources and shelter, playing a vital role in its survival and reproduction strategies.

For *P. roborovskii*, HFI, TOC, and Bio12 are the main factors influencing their habitat distribution. The distribution of *M. unguiculatus* shows a significant response to Bio12, TOC, and Bio1. Typically found in desert steppe environments, *M. unguiculatus* is well adapted to arid conditions. Previous studies have indicated that changes in annual precipitation and mean temperature can cause fluctuations in the population density of *M. unguiculatus*. Lower precipitation and extreme temperatures can lead to a scarcity of water and food resources, which in turn affects population numbers. Additionally, the soil organic carbon content, which serves as an indicator of soil quality and nutrient availability, plays a pivotal role in determining the adequacy of food resources and the overall suitability of the habitat for *M. unguiculatus*. Similarly, Bio12, Bio5, and Bio1 are key drivers affecting the distribution of *M. meridianus* in the region [46]. When the surrounding environmental temperature is too high and does not meet the biological and ecological requirements of species, it can lead to species dispersal, population decline, or even extinction. Although *M. meridianus* and *M. unguiculatus* mainly inhabit desert and semi-desert areas, significant temperature increases have a substantial impact on their distribution.

### 4.2. Impact of Climate Change on Rodent Distribution

The distribution of rodents in Ordos desert grassland exhibits significant regional differences, being more concentrated in the western and southern parts of Ordos. Differences in spatial use can effectively promote species coexistence. On a larger scale, species with similar ecological characteristics tend to select similar resource spaces to facilitate coexistence, rather than eliminating spatial competition [47]. *A. sibirica* (89%), *P. roborovskii* (68%), *M. meridianus* (63%), and *M. unguiculatus* (50%) have the largest suitable habitat areas in this region and are representative rodent species of the desert grassland habitat. Their ecological habits are similar, and their spatial distribution overlaps significantly.

Global climate change, driven by rising carbon dioxide emissions and increasing temperatures, has significantly altered the suitable habitat areas for various animal species. Against the background of global climate change, with the increase in global carbon dioxide emissions and temperatures, the suitable habitat areas for different animals have changed significantly. In this study, we observed distinct variations in the distribution of different rodent species as a result of warming temperatures. As the climate has warmed, the high-suitability areas for *A. sibirica* and *P. roborovskii* have notably decreased. This may be because these rodent species have a weaker tolerance to high-temperature environments, leading to the deterioration of their habitat conditions and a reduction in suitable living areas. The warming climate might also alter the availability of key resources, such as vegetation and water, further stressing their populations and limiting their ability to thrive.

In contrast, rodent species with a higher tolerance for warmer conditions, such as *M. meridianus* and *M. unguiculatus*, have experienced an expansion in their high-suitability areas. These gerbil species are better adapted to survival in arid and semi-arid environments, where elevated temperatures and lower moisture availability are more common. The expansion of their habitat range suggests that climate change is creating conditions favorable to these species, enabling them to colonize new areas that were previously unsuitable. The redistribution of rodent populations due to climate change is likely to have cascading effects on ecosystem dynamics.

### 4.3. Rodent Control Strategies Under Different Climate Change Scenarios

From an ecological management perspective, it is critical to focus on regions where suitable habitats for temperature-sensitive species like *A. sibirica* and *P. roborovskii* are shrinking. Strengthened monitoring efforts in these areas are essential to mitigate further human disturbance and preserve the remaining viable habitats. This can help maintain stable population densities, which are crucial for the long-term survival of these species. Conversely, in regions where habitat suitability for species like *M. meridianus* and *M. unguiculatus* is expanding, proactive ecological regulation is required. Population control measures should be implemented to prevent potential ecological imbalances. The unchecked growth of these rodent populations could lead to overgrazing, increased soil degradation, and heightened rodent pest problems, all of which could have detrimental effects on local agriculture and biodiversity.

From the perspective of social security, the plague-endemic areas in Ordos are sustained by the plague-sensitive *M. meridianus* and *M. unguiculatus*. Under the current climate model, based on the actual capture rate and in combination with the local standard of China [48], the occurrence level of *M. meridianus* in Wushen Banner has already reached a severe level (≥15.0), and in Ejin Horo Banner and Otog Banner, the occurrence level is moderate (5.1–10.0). Under the condition of climate warming, the areas of high-suitability habitats for these two gerbil species in this region are continuously expanding, which, to some extent, increases the risk of plague transmission. Therefore, to effectively respond to the plague risk brought about by climate change, it is crucial to establish a comprehensive rodent population ecological monitoring and early warning system in high-risk areas. The regular monitoring of rodent population dynamics and distribution changes can help to identify potential plague-risk areas early, implement early prevention measures, reduce the possibility of plague outbreaks, and respond in a timely fashion to the ecological challenges posed by climate change.

### 4.4. Countermeasures and Limitations

In recent years, the combined impact of climate change and routine pastoral management has significantly affected the population density of small rodents in grassland areas. In pastoral grasslands, small rodents are generally considered pests, prompting local governments to invest substantial funds annually in controlling their populations and protecting the region’s ecology, production, and living health. However, numerous studies have demonstrated that rodents are an integral and keystone species within grassland ecosystems. Whether as keystone species for conservation or as pests for management, this study is the first to predict the current and future climate-driven suitable habitat distributions of dominant rodent species in the Ordos region and to identify key influencing factors. These results are crucial for formulating scientific ecological management and control measures.

By implementing integrated management strategies based on ecological principles, we can effectively address the ecological challenges posed by climate change, ensuring the ecological safety and human habitat health of the Ordos desert grassland. This approach provides valuable references for the sustainable management of semi-desert grassland ecosystems.

However, this study has certain limitations. Although the MaxEnt model has been proven to be highly effective in determining habitat utilization and species distributions across various species and locations, it relies solely on presence data, lacking many of the complexities associated with presence–absence analysis methods. Furthermore, this study assumes that only climate will change in future periods, with other factors remaining constant. Therefore, the predicted future suitable habitat areas may still differ from actual distributions.

## 5. Conclusions

This study used the MaxEnt model to investigate the distribution of small rodents in the Ordos desert steppe of China under current and future climate conditions, exploring the ecological factors influencing their distribution. Currently, small rodents are mainly distributed in the southern and western parts of Ordos. Under the impact of climate change, the highly suitable habitat areas for *A. sibirica* and *P. roborovskii* have decreased, while the areas suitable for gerbil species have increased. NDVI, Bio12, and TOC are key ecological factors determining the habitat suitability of small rodents in the Ordos desert steppe. The Bio5, TOC, and vegetation type are important driving factors influencing the habitat distribution of *A. sibirica* in this region. HFI, TOC, and Bio12 are key factors affecting the habitat distribution of *P. roborovskii*. The distribution of *M. unguiculatus* responds significantly to Bio12, TOC, and Bio1. Bio12, Bio5, and Bio1 are key factors influencing the habitat distribution of *M. meridianu* in this region. This has the following practical significance: First, in terms of ecological protection and biodiversity maintenance, targeted strategies can be formulated according to the changes in the suitable habitats of different rodent species. The living spaces of rodent species with reduced habitats can be protected, and the impact of the population expansion of rodent species with increased habitats on the ecological balance can be closely monitored. Second, in terms of the prevention and control of rodent damages, by understanding the habitat changes and key ecological factors for gerbil species such as *M. unguiculatus*, monitoring and prevention and control measures can be carried out in advance in potential risk areas to reduce the threat to human health.

## Figures and Tables

**Table 1:** Environmental variables.

Variable (Abbreviation)	
Annual mean temperature (Bio1)	Mean diurnal range (Bio2)
Annual precipitation (Bio12)	Normalized difference vegetation index (NDVI)
Aspect	Sand
Soil bulk density (Bulk)	Precipitation seasonality (Bio15)
Elevation	Slope
Vegetation type	Soil PH
Human footprint index (HFI)	Total organic carbon (TOC)
Max temperature of warmest month (Bio5)	Precipitation of driest month (Bio14)

**Table 2:** Area of suitable habitat.

Grade	Area (km^2^)	Percentage of the Study Area
High-suitability area	16,705	20.5%
Medium-suitability area	19,887	24.4%
Low-suitability area	22,921	28.1%
Unsuitable area	26,648	30.9%

**Table 3:** Contribution of each environmental variable to Maxent modeling process.

**Factor code**	NDVI	Bio12	TOC	HFI	Aspect	Bio5	Sand	Bio2	Bio1	Bulk	Vegetation type	Slope	Bio14
**Contribution rate/%**	26.4	15.9	13.8	9.9	9.5	4.8	4.6	3.0	2.9	2.3	2.0	1.9	1.1

**Table 4:** Key environmental factors (top 3) affecting the distribution of different rodent species.

Rodent Species Name	Variable and Percentage Contribution		
*A. sibirica*	Bio2 (28.8%)	Sand (23.3%)	Vegetation type (22.0%)
*P. roborovskii*	HFI (24.9%)	TOC (23.2%)	Bio12 (20.3%)
*M. unguiculataus*	Bio12 (27.6%)	TOC (27.4%)	Bio1 (18.5%)
*M. meridianus*	Bio12 (29.0%)	bio5 (17.4%)	Bio1(13.6%)

## Data Availability

The datasets used and/or analyzed during the current study are available from the corresponding author upon reasonable request.

## Supplementary Materials

The following supporting information can be downloaded at: https://www.mdpi.com/article/10.3390/ani15050721/s1, Figure S1: Correlation analysis of nineteen environmental factor; Table S1: Result of classified bare patches and meadow.
