# Supplementary material for: Temporal and Spatial Dynamics of Rodent Species Habitats in the Ordos Desert Steppe, China
Source: Animals (Basel). 2025 Mar 3;15(5):721. doi: 10.3390/ani15050721 (PMC11899341; doi:10.3390/ani15050721)
Supplement: Supplementary file 1 [file animals-15-00721-s001.zip › animals-3445726-supplementary.pdf]

animals

MDPI

Supplementary Material

1 Supplementary Figures and Tables

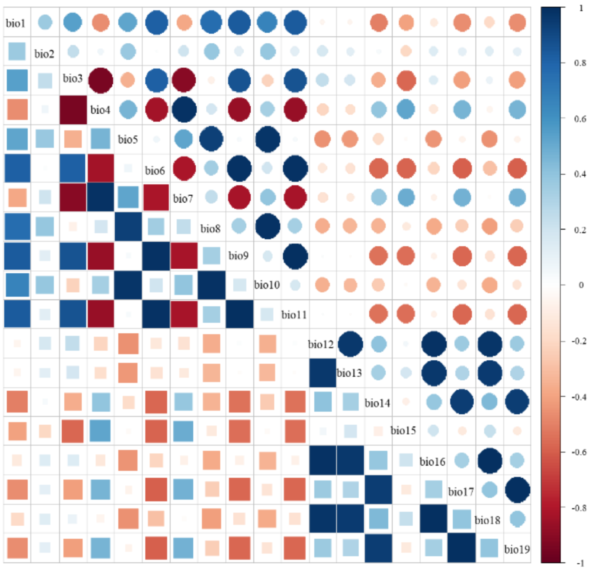

Supplementary Figure S1. Correlation analysis of nineteen environmental factor

Supplementary Table S1 Result of classified bare patches and meadow

ID	longitude	latitude
1	108.2317	38.9706
2	108.4014	39.08205
3	108.2099	39.18838
4	108.1828	39.61603
5	108.1828	39.61603
6	108.1888	39.69896
7	108.0765	39.70735
8	108.0765	39.70735
9	107.9956	39.75556
10	107.942	39.69422
11	108.6785	39.56518
12	106.856	38.47095
13	106.856	38.47095
14	107.1413	38.72025
15	107.4798	38.39813
16	107.7886	38.40393
17	107.7886	38.40393
18	107.7886	38.40393
19	107.4345	38.95787
20	107.4345	38.95787
21	107.4345	38.95787
22	107.9516	38.32159
23	107.9516	38.32159
24	107.2382	39.61787
25	107.1313	39.61659
26	107.3472	39.66423
27	107.4376	39.51218
28	107.3083	39.30016
29	107.2425	39.19718
30	107.2425	39.19718
31	107.316	39.09371
32	107.5967	39.01603
33	107.5967	39.01603
34	108.74	38.30872
35	108.74	38.30872
36	108.7529	38.30538
37	108.7529	38.30538
38	108.0632	39.36361
39	107.814	39.78554
40	107.343	39.74564

41	107.343	39.74564
42	107.5388	39.74309
43	107.5388	39.74309
44	107.3894	39.89229
45	107.6935	39.54286
46	107.3628	39.64695
47	107.6778	39.79034
48	107.6778	39.79034
49	107.7234	39.63237
50	108.7909	38.29414
51	108.7909	38.29414
52	108.7909	38.29414
53	108.7909	38.29414
54	108.1082	39.21177
55	108.1082	39.21177
56	108.1082	39.21177
57	108.2969	39.24387
58	108.2969	39.24387
59	108.7541	38.29762
60	108.7352	39.35469
61	108.7541	38.29763
62	108.7541	38.29763
63	107.4152	38.7417
64	107.4152	38.7417
65	107.4677	39.12378
66	107.4677	39.12378
67	107.2091	39.06576
68	107.3663	38.95452
69	108.2599	39.43487
70	109.6968	39.61568
71	109.4995	39.52442
72	109.5169	39.66786
73	109.5169	39.66786
74	109.778	39.30229
75	109.5184	39.62097
76	107.4332	38.93762
77	107.4332	38.93762
78	107.3481	38.82411
79	107.3515	38.74711
80	107.4341	38.76265
81	107.4341	38.76265
82	111.0254	40.20124

83	107.5424	38.8076
84	107.2912	38.54522
85	110.678	40.08424
86	107.2567	38.48796
87	107.6438	38.85726
88	107.7386	38.39775
89	107.7988	38.97762
90	107.9518	38.32141
91	107.9367	38.33257
92	107.8755	38.89031
93	111.1329	39.96599
94	111.1329	39.96599
95	108.0563	39.8412
96	108.0563	39.8412
97	108.1763	39.85449
98	108.1763	39.85449
99	108.1763	39.85449
100	108.1763	39.85449
101	108.49	39.96652
102	108.49	39.96652
103	108.49	39.96652
104	108.49	39.96652
105	109.7991	40.32028
106	109.7991	40.32028
107	109.7991	40.32028
108	109.7991	40.32028
109	110.1955	40.21066
110	107.7701	39.17152
111	107.6949	39.37658
112	107.5818	39.40752
113	108.0053	39.80721
114	108.0053	39.80721
115	109.4286	40.40984
116	109.4286	40.40984
117	109.4286	40.40984
118	109.4286	40.40984
119	107.9572	39.45867
120	108.5287	38.13351
121	108.517	38.14667
122	108.0907	39.48058
123	108.4503	38.18485
124	107.8519	39.81913

125	108.1429	39.83415
126	108.1429	39.83415
127	108.5085	39.97569
128	107.7919	38.89069
129	106.9539	38.504
130	108.0027	38.95254
131	108.0027	38.95254
132	107.95	38.92459
133	108.2095	39.27554
134	108.2096	39.27553
135	108.1632	39.302
136	107.7646	38.20238
137	107.7646	38.20238
138	107.4306	38.33075
139	107.9835	38.15842
140	107.9383	38.1799
141	107.9383	38.1799
142	111.0919	40.1069
143	110.4916	39.87816
144	110.4916	39.87818
145	110.4919	39.87811
146	109.0312	40.33281
147	109.0312	40.33281
148	109.2183	40.41486
149	109.2183	40.41486
150	109.2183	40.41486
151	109.0304	40.33307
152	109.0304	40.33307
153	109.217	40.41579
154	109.5732	39.87583
155	109.5722	39.87576
156	109.5722	39.87576
157	109.6598	39.78675
158	109.6598	39.78675
159	108.8417	38.36061
160	108.8417	38.3606
161	108.8417	38.3606
162	108.8417	38.3606
163	108.8416	38.36077
164	108.8416	38.36077
165	108.8416	38.36077
166	108.8798	38.35032

167	108.8798	38.35032
168	108.8798	38.35032
169	108.0384	39.87757
170	108.0384	39.87757
171	108.2929	39.87134
172	108.2929	39.87134
173	109.9547	39.63905
174	109.9547	39.63905
175	107.5113	38.51601
176	107.5113	38.51601
177	107.5113	38.51601
178	107.527	38.49897
179	106.9266	38.74784
180	106.9266	38.74784
181	107.8342	39.46805
182	107.8342	39.46805
183	107.8342	39.46805
184	109.2675	39.61254
185	109.2209	39.59491
186	109.2209	39.59491
